# Hematemesis after peroral endoscopic myotomy: always computed tomography-scan?

**DOI:** 10.1055/a-2740-3279

**Published:** 2025-11-26

**Authors:** Francesco Cocomazzi, Marco Gentile, Sonia Carparelli, Laura Varano, Francesco Perri

**Affiliations:** 1 577188 Division of Gastroenterology and Digestive Endoscopy, Fondazione Casa Sollievo della Sofferenza IRCCS, San Giovanni Rotondo, Italy; 2 577188 Anesthesiology and Intensive Care Medicine, Fondazione Casa Sollievo della Sofferenza IRCCS, San Giovanni Rotondo, Italy

Peroral endoscopic myotomy (POEM) is a well-established therapy for achalasia. Although considered safe, postoperative adverse events might pose a challenge for the endoscopist. Bleeding usually occurs during the procedure; conversely, a delayed one is very uncommon (0.2%), with a higher incidence in the first 72 h. Literature about its treatment is poor and limited to case reports and series 1 2 3 4 5 .

A 46-year-old man with type II achalasia underwent POEM, using a posterior approach, without complications ( Video 1 ). After 24-hours, he started drinking clear fluid asymptomatically and was discharged on the second postoperative day. A soft purée diet and esomeprazole (40 mg BID) were prescribed and taken regularly. On the eighth post-operative day, he suddenly developed dysphagia, followed a few hours later by hematemesis. Fever, tachycardia, hypotension and chest pain were all absent. No drop of hemoglobin was found. An esophagogastroduodenoscopy (EGD) revealed an occluding clot in the esophageal lumen, successfully removed, without signs of hematoma 1 2 ( Fig. 1 , Fig. 2 ). Mucosotomy clips were dislodged and an ulceration at the site was detected ( Fig. 3 ). Neither a computed tomography (CT) scan nor other procedures were performed. The patient continued fasting, proton pump inhibitors, monitoring parameters, and blood count for 24 hours, without any alterations. Then, he restarted the liquid diet. After 1 month from POEM, the patient resumed a free diet without complications.

Endoscopic treatment and complication management.Video 1

**Fig. 1:**
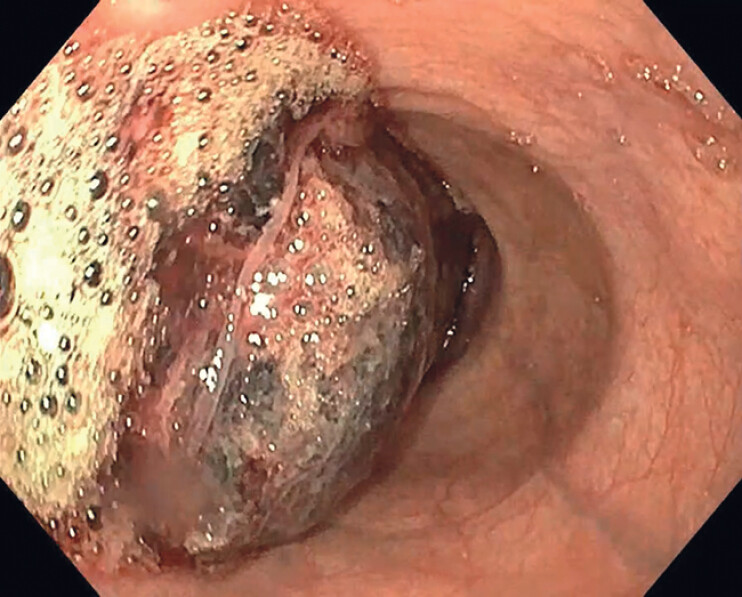
Occluding clot in the esophageal lumen.

**Fig. 2:**
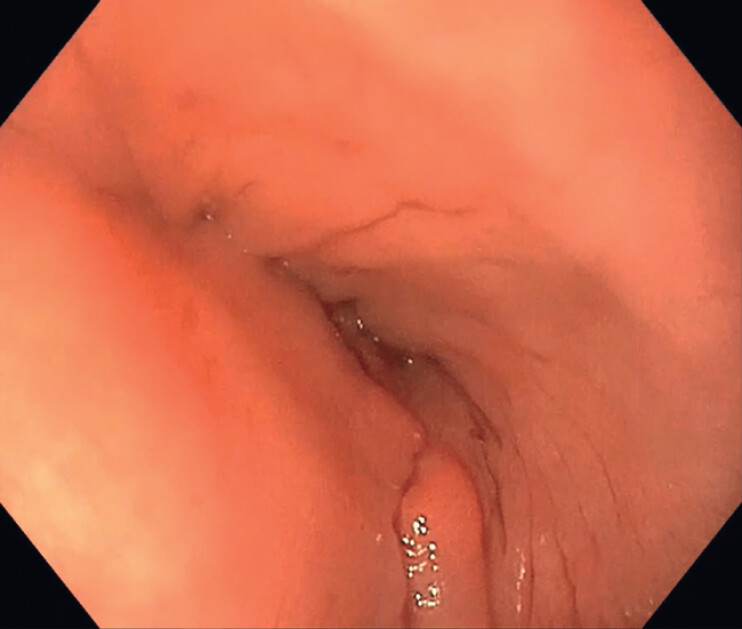
Esophageal endoscopic appearance after clot removal.

**Fig. 3:**
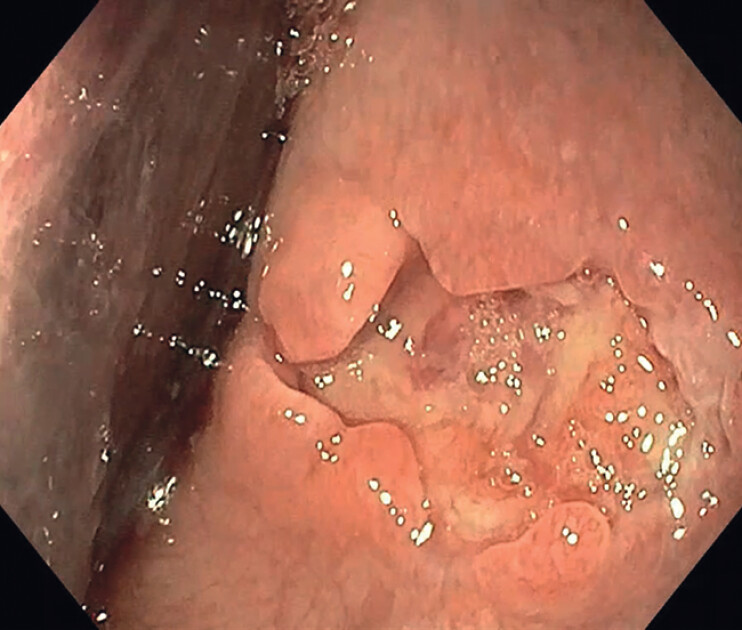
Mucosotomy findings at EGD. Abbreviation: EGD, esophagogastroduodenoscopy.

Unlike other cases 1 2 3 , our complication arose later, without the classical signs/symptoms (pain, hemodynamic alterations, and hemoglobin drop) described. Only two colleagues reported late delayed bleeding, but in symptomatic patients (hypotension, anaemia, or pain) 4 5 .

According to the current literature, our case seems unique in its presentation. His stability, the absence of other symptoms or endoscopic signs of hematoma, led us to successfully adopt a conservative management, without the need to expose this young patient to radiation of a CT scan.

In conclusion, given its rarity, also in light of what has been reported in the literature so far, the management of this complication should always be tailored to the individual patient.

Endoscopy_UCTN_Code_CPL_1AH_2AM

## Notes

Endoscopy E-Videos https://eref.thieme.de/e-videos
*E-Videos* is an open access online section of the journal *Endoscopy* , reporting on interesting cases and new techniques in gastroenterological endoscopy. All papers include a high-quality video and are published with a Creative Commons CC-BY license. Endoscopy E-Videos qualify for HINARI discounts and waivers and eligibility is automatically checked during the submission process. We grant 100% waivers to articles whose corresponding authors are based in Group A countries and 50% waivers to those who are based in Group B countries as classified by Research4Life (see: https://www.research4life.org/access/eligibility/ ). This section has its own submission website at https://mc.manuscriptcentral.com/e-videos .
